# Cerebral Venous Thrombosis after SARS-CoV-2 Infection and Pfizer-BioNTech Vaccination against COVID-19

**DOI:** 10.3390/diagnostics12051253

**Published:** 2022-05-18

**Authors:** Martina Nicolardi, Daniele Urso, Silvia Luceri, Giancarlo Logroscino, Roberto De Blasi

**Affiliations:** 1 Department of Diagnostic Imaging, Pia Fondazione di Culto e Religione “Card. G. Panico”, 73039 Tricase, Italy; lucerisilvia@gmail.com (S.L.); rdeblasi@piafondazionepanico.it (R.D.B.); 2 Center for Neurodegenerative Diseases and the Aging Brain, Department of Clinical Research in Neurology, University of Bari ‘Aldo Moro’, “Pia Fondazione Cardinale G. Panico”, 73039 Tricase, Italy; danieleurso010@gmail.com (D.U.); giancarlo.logroscino@uniba.it (G.L.); 3 Department of Basic Medicine, Neuroscience, and Sense Organs, University of Bari ‘Aldo Moro’, 70124 Bari, Italy

**Keywords:** cerebral venous thrombosis, COVID-19, vaccination, Susceptibility-Weighted Images

## Abstract

In the last 3 years, COVID-19 pandemic has produced great impacts on global population in terms of health and social costs. Pneumonia represents only one of several pathologies associated to COVID-19 disease. Among these, the cerebral venous thrombosis (CVT), constitutes an important cause of stroke. Here, we report a case of CVT diagnosed approximately 2 weeks after first dose of Pfizer-BioNTech vaccination, in a patient affected by COVID-19 few months earlier. He presented with headache and severe asthenia. The laboratory tests put in evidence thrombocytopenia and D-dimer elevation. A brain magnetic resonance imaging (MRI) and a computed tomography (CT) demonstrated hemorrhagic and ischemic phenomena on the right ventral thalamic nuclei, left thalamus, hippocampal and parahippocampal regions and the splenium of the corpus callosum. The study revealed a poorly opacified vein of Galeno and straight sinus. Heparin administration improved his clinical status; platelets values also arose over time.

**Figure 1:**
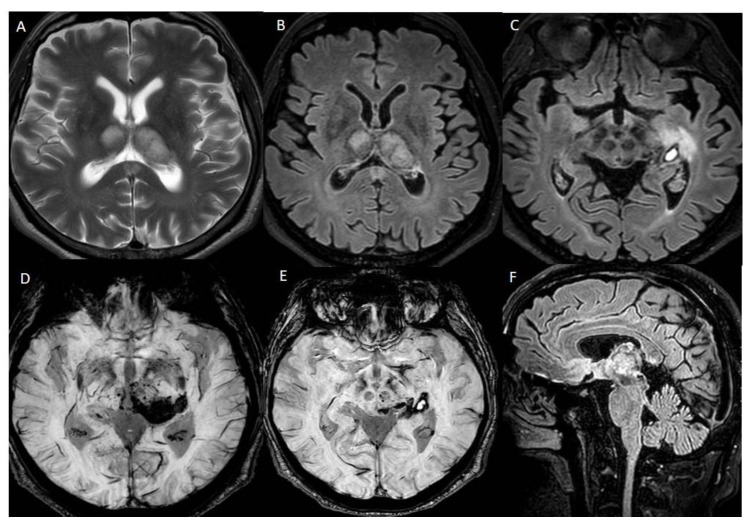
Brain MRI performed on a Philips Ingenia 3T MR system (Philips Healthcare, Best, The Netherlands), without medium contrast: (**A**) hyperintensity areas in both thalami (Axial T2-weighted image), (**B**,**C**) ischemic phenomena in thalami, left hippocampal and parahippocampal regions and haemorrhagic lesion in left parahippocampal region, view as hyperintense areas (Axial FLAIR-weighted images), (**D**,**E**) heavy metal deposits in the aforementioned regions (SWI, Susceptibility-Weighted Images), (**F**) ischemic lesion in the central portion of splenium of the corpus callosum (Sagittal FLAIR-weighted image). MRI was the first exam performed on the patient, a 56-year-old man who was hospitalized in July 2021 for severe asthenia, headache and acute confusional state, which started about 15 days after the first dose of Pfizer vaccine. The MRI sequences aforementioned allowed us to diagnose a subacute ischemic stroke. Chest X-ray, electrocardiogram and routine blood tests gave a negative response, except for high glycemic values (82 mmol/mol) and low platelets values (88 × 10^−9^/L) compared to the usual, normal patient’s platelet count. Genetic screening for coagulation disorders was negative.

**Figure 2:**
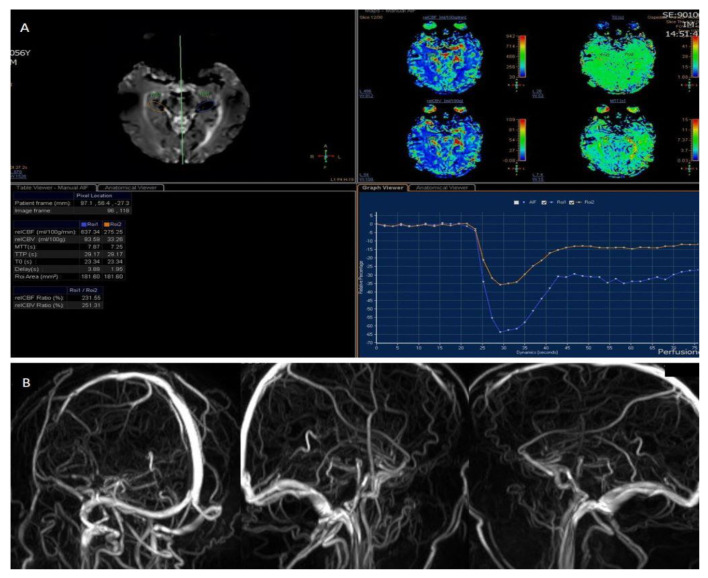
Perfusion weighted imaging executed using (**A**) Contrast Enhanced perfusion MRI (CEpMRI) revealed low levels of Cerebral Blood Flow (CBF, 275.25 mL/100 g/min) and Cerebral Blood Volume (CBV, 33.26 mL/100 g) in affected areas, compared to contralateral ones. (**B**) Phase Contrast Angiography (PCA)-MRA 3D Venography shows partial obliteration of straight sinus and lack of opacification of the vein of Galeno. CVT is a rare but considerable cause of stroke. It affects all ages but there is an increased incidence in older and young adults. The women-to-men ratio is 2.9–1.2. Some studies have shown rare cases of CVT related to COVID-19 infection and also to vaccination against it, in particular after ChAdOx1 nCoV-19 vaccine (AstraZeneca, Cambridge, UK); 1 of these was a multicentre cohort study which involved 43 hospitals across the UK [1]. Thromboembolic complications have been reported also in hospitalized patients with COVID-19, with a rate of 21–32%, due to a hypercoagulable state in those patients [2]. The most common clinical features of CVT are headache, papilledema, focal motor or sensory deficits and seizures [3]. There have been reported numerous causes of CVT, that can be categorized in infectious and non-infectious. Infectious causes include septic cranial trauma, meningitis, cerebritis, septicemia, otitis and sinusitis; among the non-infective aetiologies, malignancies, red cell and platelet disorders, coagulopathies and inflammatory disease deserve some recognition [4]. Symptoms and signs of CVT can be varied and not always typical. Neuroimaging tools play an important role in diagnosing CVT and therefore also in ensuring a timely therapeutic intervention [5].

**Figure 3:**
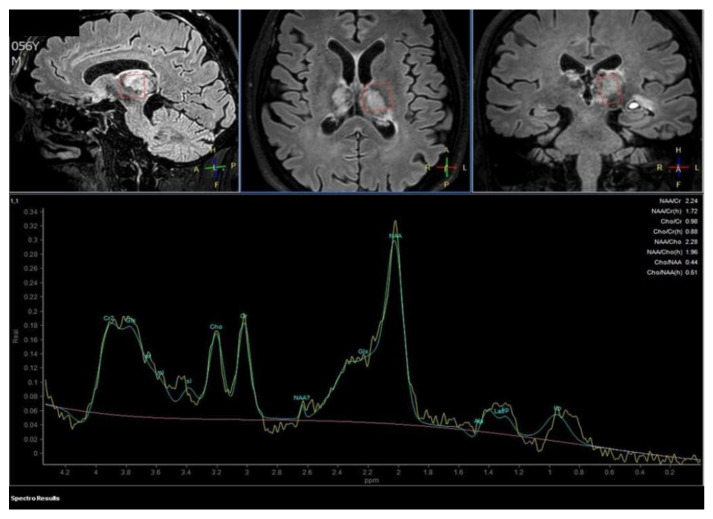
Proton MR Spectroscopy executed on the left thalamus demonstrates a normal spectrum of cellular metabolites, without anomalous metabolic peaks. The patient was soon candidate for anticoagulant therapy with Enoxaparin 6000 I.U./0.6 mL.

**Figure 4:**
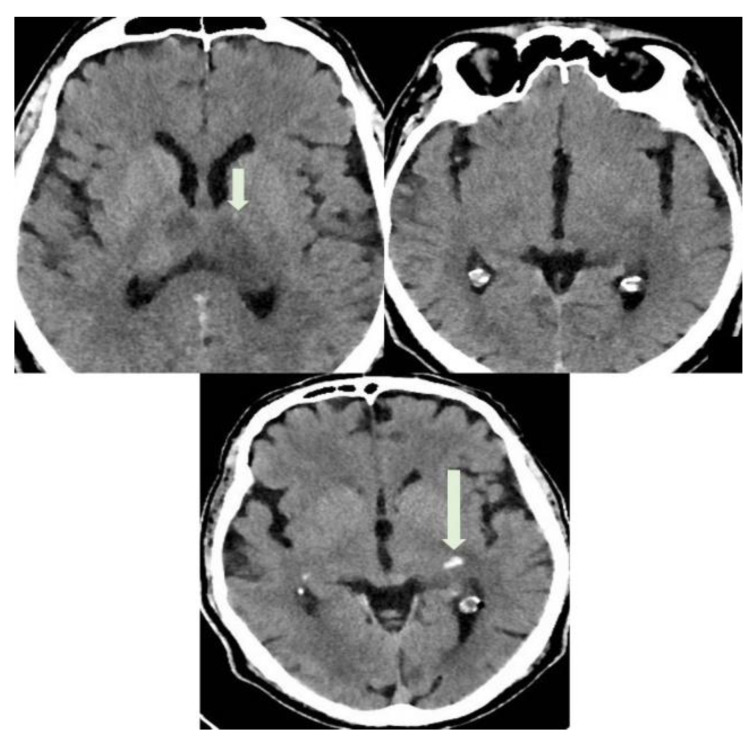
A CT with use of a medium contrast was performed the following day to better appreciate the vascular extension of the thrombosis. In the figure, axial CT images show hypodense areas in thalami and left parahippocampal region (short arrow, ischemic stroke lesions) and spontaneously hyperdense focus in left parahippocampal region (long arrow). This last was interpreted as a focal hemorrhagic transformation of the stroke. CVT in COVID-19 disease has been associated with a strong activation of the inflammatory chain. Specifically, it has been proven the implication of neutrophil extracellular traps (NETs), impaired systemic microcirculatory function, downregulation of natural anticoagulation phenomena and vascular endothelial dysfunction. Alveolar capillary microthrombi from COVID-19-induced immunothrombosis have been also described [3]. Other studies have reported CVT after vaccination against COVID-19 [6]. Thrombotic thrombocytopenia and cerebral venous sinus thrombosis have been observed in patients after receiving the adenovirus-based vaccine (AstraZeneca) and after the first dose of the mRNA-based vaccine, as in our case. Of note, a pathological mechanism which causes CVT in these cases has been postulated, known as “vaccine-induced immune thrombotic thrombocytopenia” (VITT) [7]. Low platelet count and high D-dimer values, as in our case, can assist the diagnosis of VITT after vaccination [8].

**Figure 5:**
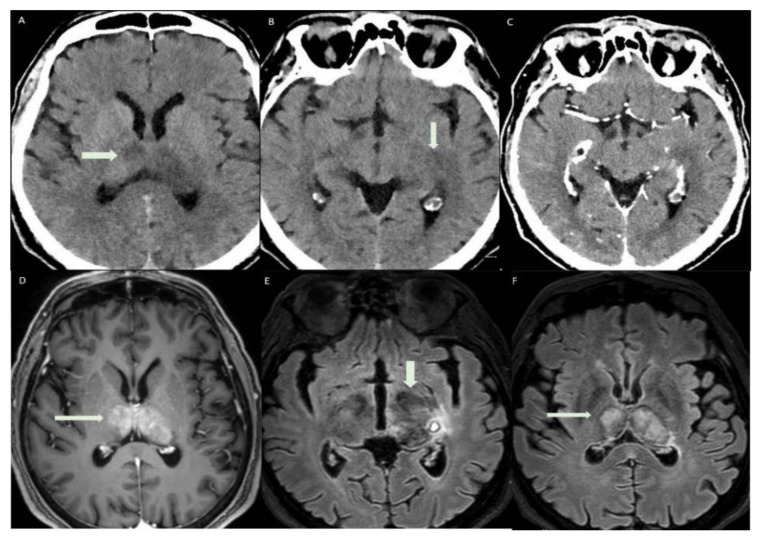
(**A**–**C**) Axial CT images show regular densitometric evolution of ischemic areas, with less hypodensity and reduction in hemorrhagic focus in the left parahippocampal region. (**D**) Axial TSE T1-weighted post contrast sequence documents reperfusion phenomena in both thalami (arrow); In (**E**,**F**), axial FLAIR-weighted sequences show a reduction in the diameter of hemorrhagic focus, compared to the first MRI exam, and less intense signal in the ischemic foci.

**Figure 6:**
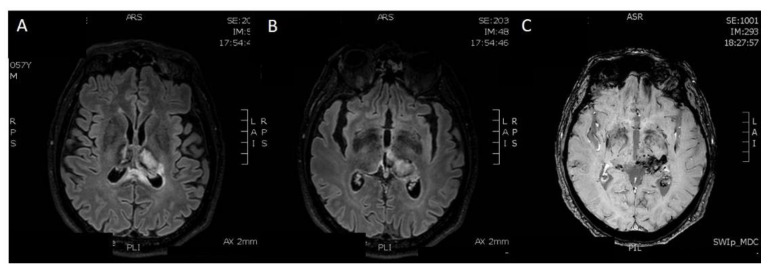
Five months later, he underwent a brain MRI exam. (**A**,**B**) Axial FLAIR-weighted sequences show reduction in the diameter of haemorrhagic focus and reduction in perilesional oedema in both thalami. (**C**) In the Susceptibility-Weighted Images, old hematic deposits can be found in both thalami, particularly on the left. Therefore, the exam showed a tendency to encephalomalacic transformation of the ischemic regions and improved sinus rectum patency, with some wall irregularities. The patient continued treatment with Enoxaparin.

## Data Availability

Data is contained within the article.
